# The Fundamental Role of NOX Family Proteins in Plant Immunity and Their Regulation

**DOI:** 10.3390/ijms17060805

**Published:** 2016-05-27

**Authors:** Ya-Jing Wang, Xiao-Yong Wei, Xiu-Qing Jing, Yan-Li Chang, Chun-Hong Hu, Xiang Wang, Kun-Ming Chen

**Affiliations:** State Key Laboratory of Crop Stress Biology in Arid Areas, College of Life Sciences, Northwest A&F University, Yangling 712100, China; yajingwang@nwsuaf.edu.cn (Y.-J.W.); weixiaoyongshengwu@163.com (X.-Y.W.); xiuqingjing@nwsuaf.edu.cn (X.-Q.J.); shizhang7026330@163.com (Y.-L.C.); ourcarrot@163.com (C.-H.H.); wangxiang1342@126.com (X.W.)

**Keywords:** NADPH oxidases, reactive oxygen species, kinases, Ca^2+^, hormones, abiotic stress, biotic stress

## Abstract

NADPH oxidases (NOXs), also known as respiratory burst oxidase homologs (RBOHs), are the major source of reactive oxygen species (ROS), and are involved in many important processes in plants such as regulation of acclimatory signaling and programmed cell death (PCD). Increasing evidence shows that NOXs play crucial roles in plant immunity and their functions in plant immune responses are not as separate individuals but with other signal molecules such as kinases, Rac/Rop small GTPases and hormones, mediating a series of signal transmissions. In a similar way, NOX-mediated signaling also participates in abiotic stress response of plants. We summarized here the complex role and regulation mechanism of NOXs in mediating plant immune response, and the viewpoint that abiotic stress response of plants may be a kind of special plant immunity is also proposed.

## 1. Introduction

Plants are frequently affected by various adverse stress factors throughout their whole life cycle. These stress factors are generally grouped into two kinds, biotic and abiotic stresses. The former mainly includes pathogens, like fungi, bacteria, viruses, nematodes, and herbivorous insects [1], causing massive losses to global agriculture. The latter includes heat, cold, drought, salinity, light, water, ozone, heavy metals, UV radiation, and other factors [2], and statistics indicate that it reduces overall yields of staple crop plants by more than 50% [3]. Animals can move from place to place when they encounter dangerous environments, but plants can not move away when they are subjected to environmental stresses. On the basis of this, plants have developed specific strategies that protect them from defective and complex stress conditions.

In the traditional sense, plant immunity refers to the biotic stress response. During biotic stresses, in addition to the external barriers of plant cells wall, the internal innate immune system shows a complex defense signaling network that plants use to cope with microbial threats. Firstly, the surface-localized pattern recognition receptors (PRRs) recognize the pathogen-associated molecular patterns (PAMPs), which triggers the first line of plant innate immune system and is termed PAMP triggered immunity (PTI) [4]. However, to achieve more effective infection, many pathogens have acquired the ability to inject virulence effector proteins into host cells, further dampening the host immune systems or interfering with host physiological and cellular responses [5]. The intracellular immune receptors that are most often nucleotide-binding domain and leucine-rich repeat-containing receptor (NLR) proteins can recognize those effectors and elicit a second layer of defense defined as effector-triggered immunity (ETI) [6].

A common feature of plant response to both biotic and abiotic stresses is the burst of the so-called reactive oxygen species (ROS), which include singlet O_2_, hydroxyl radical (OH^−^), hydrogen peroxide (H_2_O_2_), superoxide radical (O_2_^−^), *etc.* ROS play a central role in the defense process of plants [7]. For example, ROS can induce plants to generate defense molecules that arrest pathogen growth [8]. It was reported that abscisic acid (ABA) is closely related to a broad range of stress responses of plants and the ABA-induced ROS production causes stomatal closure, increasing resistance to penetration by pathogens and at the same time decreasing water loss under drought conditions [9]. ROS promote the establishment of systemic acquired resistance (SAR), a type of long-distance signaling response following exposure to pathogens [10]. However, the function of ROS is dependent on their intracellular concentration. Numerous studies have shown that low-concentration ROS can act as signal molecules mediating the regulation of plant acclimatory signaling in the stress response, whereas high-concentration ROS can destroy cellular redox state equilibrium, result in damage to chemicals, and then change the state of cell metabolism and induce programmed cell death (PCD) [11,12,13]. ROS are involved in ETI-induced hypersensitive response (HR) in plants, a PCD process that promotes cell death around the infection site, which limits the spread of the pathogens [14] and restricts the amount of cell death in response to pathogen recognition [15]. However, it is also important for plants to scavenge redundant ROS so as to prevent excess induction of PCD. Because stress-induced PCD significantly affects plant yield and productivity, it is therefore of fundamental importance in agriculture [16]. All these results suggest that ROS production is of vital importance in plant immunity.

Many studies have shown that the plasma membrane-localized NOXs are major ROS producers of plants under normal and stress conditions [17,18]. They produce O_2_^−^ in the apoplastic space, and then the produced O_2_^−^ can be converted to H_2_O_2_ by superoxide dismutase [11,12,13]; the H_2_O_2_ diffuses into the cytosol. Several different mechanisms have been found to be involved in apoplastic ROS sensing and downstream reactions in plants [19] and apparently, as the major ROS producers, NOXs might play vital roles in plant immunity. Understanding the role and regulation mechanism of NOXs in plant immunity could help us protect plants from adverse effects resulting from excess ROS production under various environmental stress conditions. Here, we fully discuss how the NOXs interact with signaling molecules in plant immunity, and the viewpoint that abiotic stress response may be a part of plant immunity is also proposed.

## 2. NOXs Participate in Plant Immunity

**Figure 1:**
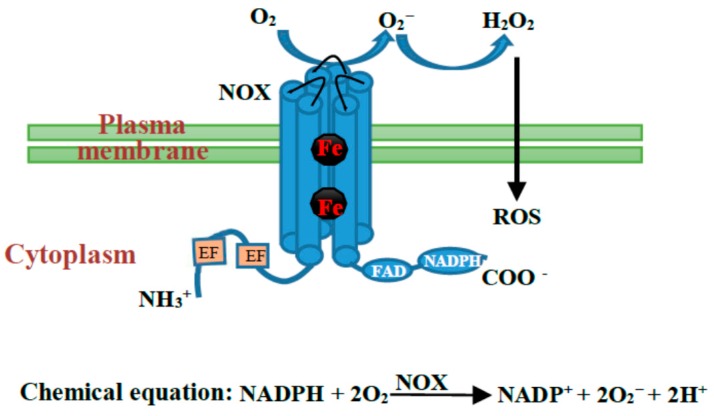
The schematic representation of NADPH oxidase (NOX) proteins in plants. The plant NOX proteins are often composed of a six-transmembrane domain, two hemes, a C-terminal flavin adenine dinucleotide (FAD) and NADPH hydrophilic domain, and two N-terminal EF-hand motifs. The NOX proteins obtain electrons from the cytoplasmic electron donor NADPH, and then transfer the electrons through the membrane to the extracellular electron acceptor O_2_ to generate O_2_^−^.

NOX, called respiratory burst oxidase (RBO) in mammals, was first identified to function in mammalian ROS production, and total seven types of NOXs, namely NOX1, NOX2, NOX3, NOX4, NOX5, and two dual oxidases (DUOX1 and DUOX2) were identified in animals [20,21]. All the animal NOX/DUOX proteins contain a six membrane-spanning domain, two hemes, and a conserved domain involved in NADPH and flavin adenine dinucleotide (FAD) binding. In addition, NOX5 also contains four calcium-binding EF-hand motifs in its N-terminus while DUOX proteins also contain two EF-hand motifs and an additional transmembrane domain (a peroxidase-like domain) in their N-terminus [21]. NOXs in plants are RBO homologs (RBOHs) [17,18]; however, only NOX5-like homologs have been found in plants, even though multiple members exist in different species [18,21,22]. The first studied NOX gene in plants was *Oryza sativa* *OsRbohA* [23]. Since then, NOX genes were identified and cloned in tomato [24], tobacco [25], potato [26], *Arabidopsis thaliana* [18], *Medicago truncatula* [27], *Phaseolus vulgaris* [28] and maize [29]. The plant NOX proteins are often composed of a six-transmembrane domain, two hemes, a C-terminal FAD and NADPH hydrophilic domains, and two N-terminal EF-hand motifs [18] (Figure 1). The EF-hand is defined by its helix-loop-helix secondary structure as well as the ligand presented by the loop to bind the Ca^2+^. In rice, the NOX proteins share from zero to three EF-hand motifs among the typical nine homologs [30]. The rice NOX, OsRbohB, not only has two EF-hand motifs but also has two EF-hand-like motifs; however, Ca^2+^ only binds to the first EF-hand motif [31]. In addition, using protein kinase inhibitor treatment and a quantitative phosphoproteomics method, it was revealed that multiple different phosphorylation sites exist in the plant NOXs, for example, the potato StRbohB and *Arabidopsis thaliana* AtRbohD proteins [32,33,34]. In spite of the similar structural characteristics, plant NOXs show different numbers of isoforms among the different species. For example, *Arabidopsis thaliana* has ten NOX proteins from AtRbohA to AtRbohJ [35] and eight ancient forms from ferric reduction oxidase 1 (AtFRO1) to AtFRO8 [36], whereas rice possesses nine typical NOXs (OsNOX1–9) and only two OsFRO1 and 7 [30,37]. To illuminate the phylogenetic relationships among members of FRO and NOX family in plants, we generated an unrooted maximum-likelihood phylogenetic tree stemming from 50 FROs and 77 NOXs identified from 20 species of plants [22]. In the maximum-likelihood tree, the FRO and NOX homologs in plants can be classified into four subfamilies, namely NOX, FRO I, FRO II, FRO III, and based on their structure characteristics, the members of FRO I, FROII, FROIII could be considered as ancestor NOXs [22].

The roles of NOXs in plant immunity are relatively more studied in *Arabidopsis* and rice. It has been shown that two *Arabidopsis* NOX genes *AtRbohD* and *AtRbohF* can control many cellular processes in pathogen defense [5,6,17]. Plants deficient in *AtRbohD* and *AtRbohF* generate less H_2_O_2_ and are shown to be more susceptible to pathogens than wild-type plants [25,38]. The stomatal closure of guard cells is also impaired in the mutants [26]. By using suspension cells, Yoshie *et al.* (2005) found that two rice NOX genes, *OsRbohA* and *OsRbohE*, participate in ROS-dependent immune responses [38,39]. In addition, inoculation of rice with yellow pathogenic bacterium strain PXO99 can improve the expression levels of two rice NOX genes, *OsRbohA* and *OsRbohB* [40,41], further suggesting that the NOX genes take part in plant immunity.

**Figure 2:**
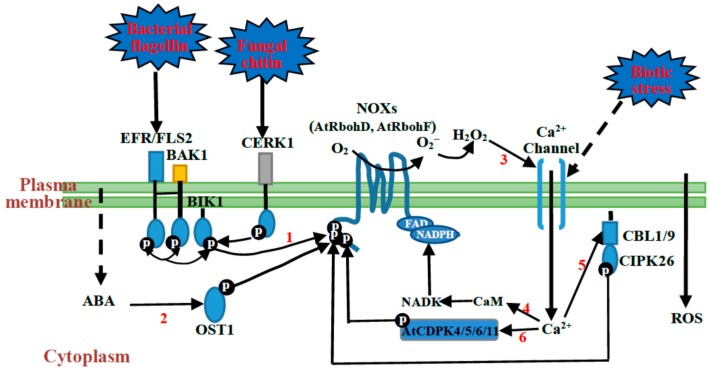
Phosphorylation-dependent regulation of NOXs during plant immunity. (**1**) Upon PAMPs perception, PRRs (pattern recognition receptors), such as chitin-elicitor receptor kinase 1 (CERK1), elongation factor-Tu receptor (EFR) and flagellin sensing 2 (FLS2), and co-receptor brassinosteroid insensitive 1 associated receptor kinase 1 (BAK1), directly phosphorylate and activate botrytis-induced kinase 1 (BIK1). Phosphorylated BIK1 has a higher binding affinity for AtRbohD and phosphorylates it on some specific sites; (**2**) In addition, the stress-mediated perception of ABA (abscisic acid) leads to activation of OST1 (open stomata 1), then it phosphorylates AtRbohF; (**3**) The produced H_2_O_2_ itself may trigger further activation of Ca^2+^ channel(s), forming a positive feedback regulation; (**4**–**6**) At the same time, a central second messenger Ca^2+^ activates CDPKs (calcium-dependent protein kinases) and CBLs (Calcineurin B-like), then they phosphorylate AtRbohD and AtRbohF, respectively. CaM (calmodulin) is also activated by Ca^2+^, then CaM regulates NADK (NAD kinase) to produce NADPH. The solid lines and dashed lines represent determinate and potential interactions, respectively.

**Figure 3:**
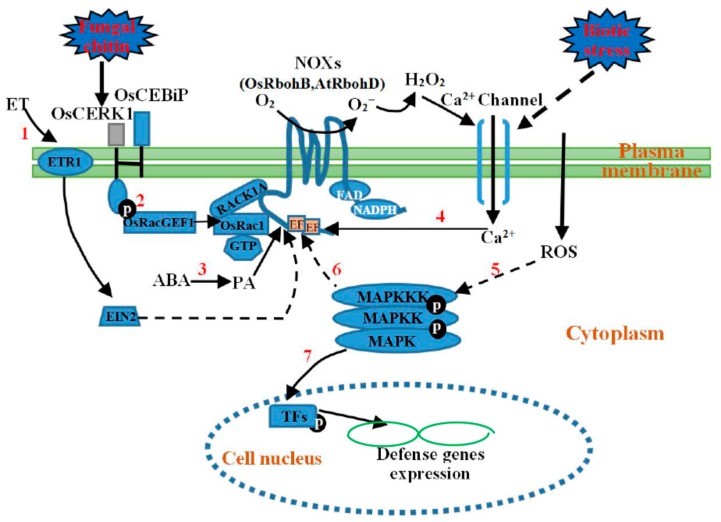
Phosphorylation-independent regulation of NOXs during plant immunity. (**1**) ET (ethylene) receptor 1 (ETR1)-and ethylene insensitive 2 (EIN2)-mediated signaling also activates AtRbohD; (**2**) Upon PAMP perception, PRRs in rice, such as chitin elicitor-binding protein (OsCEBiP) and OsCERK1, function as the receptor complex. OsCERK1 directly phosphorylates and activates OsRacGEF1, leading to GTP binding to OsRac1; at the same time, OsRac1 also interacts with the N-terminus of the OsRbohB directly or via the RACK1A (receptor for activated C-kinase 1 A) protein; (**3**) ABA induces the production of PA (phosphatidic acid), which binds to and activates AtRbohD; (**4**) Cytosolic Ca^2+^ binds to EF-hand motifs of NOXs, which leads to activation of NOXs. The produced H_2_O_2_ itself may trigger further activation of Ca^2+^ channel(s); (**5**–**7**), MAPK cascades serve as positive regulators of NOXs and produced ROS also activates MAPK cascades, ending in MAPK cascades activating or inhibiting some transcription factors (TFs) by phosphorylation to induce defense gene expression. The solid lines and dashed lines represent determinate and potential interactions, respectively.

A large number of studies have shown that the functions of NOXs in plant immunity come as the result of their extensive interactions with other immunity signaling molecules including Rac/Rop small GTPases (like rice OsRac1) [42,43], hormones (like ABA and ethylene (ET)) [44,45,46], and kinases (like receptor like kinases (RLKs), receptor-like cytoplasmic kinases (RLCKs), calcium-dependent protein kinases (CDPKs), mitogen activated protein kinase (MAPK) cascades, and open stomata 1 (OST1)) [44,46,47,48,49,50,51]. These signaling molecules were found to be tightly involved in plant immune responses from pathogen perception to gene expression regulation. Interestingly, these signaling molecules have been demonstrated to be also involved in activation of NOXs. In fact, the structural feature of NOXs provides the basis for the interaction of the proteins with these immunity signaling molecules [48,49,52,53,54]. A large number of experiments have shown that some kinases could phosphorylate NOXs directly [32,33,34,44]. According to these findings, the interaction of NOXs with these signaling molecules can be divided into two ways, the phosphorylation-dependent way (Figure 2) and phosphorylation-independent way (Figure 3); the two ways work together to promote the activation of NOXs in plants.

## 3. Phosphorylation-Dependent Regulation of NOXs during Plant Immunity

Protein phosphorylation is the most important molecular mechanism during the responses of the cells to external and internal signals when plants are growing under both normal growth and environmental stress conditions. It is well known that protein phosphorylation is the most common and important mechanism to control protein vitality. For example, MAPK cascades activate or inhibit some TFs by phosphorylation, which have an effect on the expression of target genes [55]. The function of NOXs in plant immunity is also regulated by phosphorylation. The existing studies have shown that many kinases, such as RLCKs, Ca^2+^-regulated kinases and OST1, can phosphorylate NOXs, and therefore play important roles in plant immunity.

### 3.1. Receptor-Like Kinases (RLKs)-Receptor-Like Cytoplasmic Kinases (RLCKs) Complexes-Mediated Phosphorylation of NOXs

RLKs include three major domains that are an extracellular domain, a transmembrane domain, and an intracellular kinase domain, whereas RLCKs only possess a cytoplasmic kinase domain [56,57]. It is now clear that RLKs are key PRRs for recognition of PAMPs from pathogens, while some RLCKs often functionally and physically associate with RLKs to relay intracellular signaling via transphosphorylation events [58], and then induce downstream immune responses [59]. Among these responses, RLKs or RLCKs can interact with NOXs by directly or indirectly phosphorylating the proteins for transmitting pathogen signals during plant immunity [52,60].

It was found that one NOX protein, AtRbohD, fuctions as the key resource of ROS during plant immunity in *Arabidopsis*, and botrytis-induced kinase1 (BIK1), a protein of RLCKVII subfamily members, can phosphorylate AtRbohD to produce ROS in the plant [52]. At the same time, BIK1 was found to directly bind to multiple RLKs PRRs in the resting state, such as flagellin sensing 2 (FLS2), elongation factor-Tu receptor (EFR) and chitin-elicitor receptor kinase 1 (CERK1) [61,62,63]. These three proteins are the most widely studied PRRs in plant immunity, and they can specifically perceive a conserved 22 amino acid peptide of bacterial flagellin (flg22), a conserved N terminal peptide sequence of the bacterial elongation factor-Tu (termed elf18) and chitin, respectively [61,62,63], leading to activation of a series of immune responses that culminate in slowing or halting of pathogen proliferation [61,62,63]. Upon flg22 or elf18 perception, FLS2 or EFR rapidly associates with co-receptor BAK1, and then induces phosphorylation of both proteins (FLS2 and BAK1) to initiate the downstream responses [64,65]. Then, BIK1 interacts with the two RLKs, FLS2 and BAK1, to be rapidly phosphorylated. The phosphorylated BIK1 then directly phosphorylates AtRbohD [63] to produce ROS. Further proteomic analyses and kinase assay revealed that BIK1-specific phosphorylation sites are located in the residues of Ser39, Ser339, Ser343 and Ser347 within the N-terminal part of AtRbohD, and the mutations in these phosphorylation sites could suppress the PAMP-triggered ROS bursts in the plants [47]. Because of the high conservation of NOXs in structure among different species of plants [32,37], the residues phosphorylated of NOXs by BIK1 found in *Arabidopsis* may also occur in other species.

Not only BIK1, but also other RLCKs participate in activation of NOXs in plants. It was found that Brassinolide-signaling kinase 1 (BSK1) and PBS1-like 1 (PBL1), which belong to the subfamily RLCK-XII and VII, respectively, can also associate with FLS2 *in vivo* in *Arabidopsis* [61,62,66]. In rice, a RLCK, namely OsRLCK185, can interact with OsCERK1 *in vivo* [67], implying that it may also participate in the activation of NOXs. In addition, other RLCKs, such as PBL2 and PBL5, were found to be genetically required for the full PAMP-induced ROS production [62,63]. More recently, we found that the levels of transcripts of three RLKs, namely OsRPK1, OsRPK2 and OsRPK3, were correlated with the level of *OsRbohA* transcripts in rice [30], indicating that these OsRPKs might be essential for the function of OsRbohA in the plant defense response. However, further study is needed to determine whether the mechanism also depends on the phosphorylation of OsRbohA by these three RLKs.

Different from these PAMP-triggered PTI, ETI is specifically induced by the interaction of intracellular disease resistance (R) proteins and cognate effectors produced by pathogens [6]. For instance, resistance to pseudomonas syringae 5 (RPS5), one intracellular R protein, functions as a guard to monitor bacterial effector AvrPphB [68]. It is well known that R proteins are most often NLR proteins that are imported into the nucleus where they are apparently active, suggesting that R proteins may function in the nucleus apart from the cytoplasm [69,70,71]. ROS production is one of the earliest responses, starting only a few minutes after PAMP treatment, while the production of ROS during ETI occurs at a much slower pace [72]. Thus, it is possible that R proteins first bind to targeted defense genes that then have an effect on the regulation of NOXs in infected cells, thus slowing the rate of ROS generation. However, the regulatory mechanisms of NOXs in ETI signaling remain unclear. However, some studies have shown that plants have established a positive relationship between ETI and PTI through BIK1 during bacterial infection [62]. The aforesaid bacterial effector AvrPphB can interact with BIK1 and then proteolytically cleave BIK1, whereas RPS5 can detect the changes in BIK1 and contribute to its functioning in the regulation of AtRbohD [62].

### 3.2. Ca^2+^-Regulated Kinases-Mediated Phosphorylation of NOXs

Ca^2+^ is a central secondary messenger in plants, contributing to a plethora of signaling responses. An increase in cytosolic Ca^2+^ concentration usually occurs as early event in plant-pathogen interactions, then the stimuli is transduced to intracellular responses [73]. Patch clamp studies suggested that a cell membrane Ca^2+^-permeable ion channel can be activated to conduct Ca^2+^ afflux inwardly by elicitors during pathogen infection in plants [74]. In addition to the extracellular pool functioning as a Ca^2+^ source, the internal stores, such as the endoplasmic reticulum and vacuoles, are gaining appreciation [75].

Ca^2+^ exerts three important functions in the process of NOX-mediated signaling in plant immunity. Firstly, NOXs have Ca^2+^-binding EF-hand motifs in their N-terminal regions and thus Ca^2+^ can directly regulate the activity of NOXs and therefore participate in ROS production in plants [76]. Secondly, Ca^2+^ works together with its binding proteins in the regulation of NOX activity. Plants are endowed with two principal classes of Ca^2+^-regulated kinases. The first class is composed of CDPKs [77]. Experiments based on quantitative phosphoproteomics and selected reaction monitoring tandem mass spectrometry revealed that multiple different Ser residues in the N-terminal region of AtRbohD were phosphorylated in response to PAMP stimulation in *Arabidopsis* [33,34]. Four CDPKs, namely AtCDPK4, AtCDPK5, AtCDPK6 and AtCDPK11, were identified as positive regulators of AtRbohD after flg22 treatment [48]. A further study proved that AtCDPK5 is involved in the phosphorylation of Ser39, Ser148, Ser163 and Ser347 of AtRbohD [53]. In addition, it was found that NtCDPK2VK, the constitutively active mutant of tobacco NtCDPK2, could induce ROS production, while StCDPK5VK, the constitutively active mutant of potato StCDPK5, could phosphorylate StRbohB in *Nicotiana benthamiana* leaves [78,79]. These results suggest CDPK-dependent phosphorylation occurs widely in different species in the regulation of NOX activity. The second class of Ca^2+^-regulated kinases is represented by Calcineurin B-like (CBL)-interacting protein kinases (CIPKs) that become activated upon interaction with CBL Ca^2+^ sensor proteins [80,81]. A recent report showed that calcium sensor CBL10 and its interactor protein kinase Cipk6 contribute to ROS generation during PTI and ETI in the interaction of *Pseudomonas syringae* pv tomato DC3000 and *Nicotiana benthamiana* [82], which may work through direct phosphorylation of NbRbohB. Additionally, in *Arabidopsis,* CIPK26 interacts with the plasma membrane-localized Ca^2+^ sensors CBL1 and CBL9; they work together to phosphorylate AtRbohF by CIPK26 interaction with the N-terminus of AtRbohF [83]. Many studies have shown that CBL-CIPK complexes contribute to the tolerance of plants to various abiotic stresses such as salt, cold, and drought [84,85,86]. It is possible that CBL-CIPK complexes participate in regulation of NOXs; therefore, they function in plant stress tolerance. Thirdly, Ca^2+^ indirectly regulates the activity of NOXs in plants by binding to calmodulin (CaM). A previous study reported that three types of tobacco CaM isoforms participate in the activation of NAD kinases (NADKs) [87]. NADKs are the enzymes found in both prokaryotes and eukaryotes. They generate the important pyridine nucleotide NADPH/NADP from substrates ATP and NADH/NAD [88] and therefore regulate the activity of NOXs. The evidence now accumulating suggests that ROS and other free radicals can activate Ca^2+^-permeable channels in the plant plasma membranes causing Ca^2+^ elevation in the cytosol [17]. It seems that NOXs, ROS, Ca^2+^ and Ca^2+^-regulated kinases can form a signaling loop in the plant stress response.

### 3.3. Open Stomata 1 (OST1)-Mediated Phosphorylation of NOXs

NOXs also participate in the regulation of stomatal movement in plants. It was reported that NOX-dependent ROS production in guard cells plays an important role in ABA-mediated stomatal closure [40], and several lines of evidence show that ABA-induced ROS accumulation originates from two NOX proteins, AtRbohD and AtRbohF, during stomatal closure [9]. Stomatal closure in guard cells is a basic defensive strategy of plants to prevent biotic and abiotic stresses. OST1 is a member of the sucrose non-fermenting 1 (SNF1)-related protein kinase 2 family (SnRK2s), and a mutation in the *OST1* gene impairs ABA-triggered ROS production in guard cells, suggesting that OST1 acts upstream of NOX in this signaling cascade [54]. In addition, flg22 treatment could induce stomatal closure in wild type plants but not in the *ost1* mutant in *Arabidopsis* [89], and further experiments proved that OST1-mediated ROS generation in guard cells involves the phosphorylation of AtRbohF by OST1 [44]. In this process, ABA can be perceived by the pyrabactin resistance protein 1 (PYR1), the resulted PYR1 receptor complex then leads to suppression of protein phosphatase 2Cs (PP2Cs), which function as negative regulators of OST1 [90]. The OST1-mediated phosphorylation of AtRbohF mainly occurs on Ser13 and Ser174 of the NOX protein but Thr91 and Ser97 of the protein might also be phosphorylated during the signaling transduction [44]. Considering the highly conserved serine residues on other NOX proteins, it is reasonable to believe that they can be phosphorylated by OST1 and/or other members of the SnRK2 family kinase proteins in the regulation of stomatal movement of plants.

## 4. Phosphorylation-Independent Regulation of NOXs during Plant Immunity

Although phosphorylation-based regulation is required for the activation of NOXs, other phosphorylation-independent regulation probably exists. Indeed, studies have shown that MAPK cascades, Rho-type GTPases, and hormones also tightly participate in the activation of NOXs during plant defense responses.

### 4.1. MAPK Cascades-Mediated Regulation of NOXs

MAPK cascades are one of the most important and highly conserved signaling cascades, which consist of three tier components, MAPKKKs, MAPKKs, and MAPKs, carrying out phosphorylation reactions from upstream receptors to downstream targets. For instance, MAPK cascades activate or inhibit some specific TFs by phosphorylation [91], and thus regulate the expression of many defense genes in plant stress responses. TFs transmit the signals to the nucleus where the downstream target genes are transcriptionally regulated via interaction with the cis-acting elements on the promoters of the genes. Downstream targets of MAPK cascades include many kinds of TFs, for example, those in the WRKY family, TGA transcription factors, and hormone response factors such as ethylene insensitive 3 (EIN3) [92]. Interestingly, apart from TFs acting downstream of MAPK cascades, an increasing number of studies have shown that MAPK cascades also serve as positive regulators of NOXs for ROS production and the produced ROS in turn activates MAPK cascades [49,50,51]. Despite the fact that MAPK cascades are involved in most of the signaling pathways through the phosphorylation reaction, the mechanism of MAPK cascade functioning in NOX activation is not yet clear. No direct evidence shows that NOX proteins could be phosphorylated by MAPKs. Therefore, in this stage we presumably classified the mechanism as being non-phosphorylated.

It has been found that two MAPKKs, MEK1 and MEK2, can promote NOX-derived ROS production in *Nicotiana benthamiana* during the plant immune response [49]. In *Arabidopsis*, a MAPKKK, namely MEKK1, which initiates a signaling of MEKK1-MKK4-MPK3/6, was found to act as upstream of NOX, stimulating H_2_O_2_ production in pathogen attack, and the resulted H_2_O_2_ in turn activates MPK3 and MPK6 in leaf cells of the plant [50]. In maize, a 46-kDa MAPK (p46MAPK) was found to positively regulate NOX for H_2_O_2_ production, and similarly, the produced H_2_O_2_ in turn activates p46MAPK as well [51]. It is well known that both abiotic stress factors such as salt, cold, wound, and drought, and biotic stress factors like bacterial and fungal elicitors, can activate MEKK1, but the followed MAPK cascades of MEKK1-MKK2-MPK4/6 and MEKK1-MKK4/5-MPK3/6 operate separately in the downstream regions of ROS signaling during abiotic and biotic stress responses of plants [93,94,95]. However, during the ABA-mediated stress response, MAPK cascades may act both upstream and downstream of the ROS production. For instance, a study in maize revealed that ABA activates a 46-kDa MAPK that acts downstream of H_2_O_2_ and further positively regulates NOX for H_2_O_2_ production [51]. Therefore, the interaction between NOXs and MAPKs forms a feedback adjustment process by ROS production in plant immunity. However, the mechanism for the feedback regulation remains to be elucidated.

### 4.2. Rho-Type GTPases-Mediated Regulation of NOXs

Rho-type GTPases belong to the Rat sarcoma (Ras) superfamily of small GTP-binding proteins, and plants have a sole subfamily of Rho-type GTPases, called ROPs (Rho of plants) or RACs (for the sequence similarity they share with animal Racs, a Rho subfamily). The Ras superfamily serves as two-state molecular switch depending on its GDP- or GTP-bound conformation [96]. Guanine nucleotide exchange factor (GEF) enhances the release of GDP from Rac/Rop, thereby promoting the binding of GTP, and GEFs typically exert their actions in large molecular complexes linking RLKs to the activation of small GTPases [97,98,99]. A good deal of evidence has shown that ROP activity is correlated with NOX-catalyzed ROS accumulation during polar root hair and pollen tube growth [98,99]. For example, in *Arabidopsis*, it was found that RopGEF interacts with the receptor kinase FERONIA, functioning as an upstream regulator of ROP GTPase signaling during polar root hair development [98] while FERONIA-related ANXUR receptor-like kinases, ANXUR1 and 2, may activate ROP GTPases through RopGEFs during pollen tube growth, preceding the activation of the NOX-catalyzed ROS accumulation [99].

The roles of the Rac/Rop small GTPases in plant innate immunity have also been studied in rice, barley and other species [42]. For instance, rice OsRac1 regulates cell death, stimulates expression of pathogenesis-related (PR) genes and production of phytoalexins [100,101]; these processes may be constructed by activating ROS production. The most typical example of RLK/GEF/Rho-type GTPases/-mediated regulation of NOX in plant immunity was found in chitin signaling [60]. Chitin is one of the best-characterized PAMPs in pathogenic and non-pathogenic fungi. There are two PRRs in rice, OsCEBiP and OsCERK1. OsCEBiP is a receptor like protein (RLP), it can directly bind to chitin, whereas OsCERK1 is a RLK, it does not directly bind to chitin. However, the two immune proteins could form a receptor complex to transduce the chitin signals to the downstream components during the resistance of rice plants to fungal infection [102,103]. More recently, it was found that an OsCEBiP/OsCERK1-OsRacGEF1-OsRac1 module participates in the immunoresponse in rice [60]. OsRacGEF1 can be directly phosphorylated at Ser549 after chitin treatment by OsCERK1; this leads to the activation of OsRacGEF1 and then boosts the binding of GTP to OsRac1 [60]. The activated OsRac1 then directly interacts with the N-terminus of OsRbohB and thereby stimulates ROS generation [43]. In addition, rice contains two receptors for activated C-kinase 1 (*RACK1*) genes, *RACK1A* and *RACK1B* [104]. It has been found that RACK1A can interact with the GTP form of Rac1, as well as with the N-terminal region of OsRbohB [104] and therefore contributes to ROS production and defense gene expression in rice cells. Obviously, as discussed above, the interactions between ROPs and NOXs occur under both normal plant development and the defense response.

### 4.3. Hormone-Mediated Regulation of NOXs

There is now a substantial body of literature concerning hormones that participate in plant immunity, such as ABA, jasmonic acid (JA), salicylic acid (SA), ET, *etc.* [10,45,46,105]. Intrinsic to their participation in plant immune is the interplay between ROS and these hormones, as well as hormone-dependent ROS balance through the regulation of NOX activity and ROS-scavenging capacity. For example, as a part of the complex signaling cascades of ABA-induced stomatal closure in guard cells, phospholipase D (PLD) produces the second messenger phosphatidic acid (PA), which binds to and activates AtRbohD [45]. In addition, an enhanced expression of genes associated with ET synthesis and signaling as well as a high level of AtRbohD-derived ROS accumulation was found in *Arabidopsis* after pathogen treatment [106]. The results obtained from the analysis on ethylene-insensitive mutants and *atrbohD* after flagellin treatments revealed that an ET receptor 1 (ETR1)- and ethylene insensitive 2 (EIN2)-mediated signaling is required for flagellin-induced AtRbohD-dependent ROS accumulation [46]. ET not only potentiates the accumulation of ROS, but together with ROS, also regulates cell death during plant stress responses [107]. Thus, when plants are exposed to environmental stimuli, ET and ROS may form an amplification loop that mediates cell death. ROS also plays a crucial role in SA-mediated cell death during pathogenesis responses, and in turn, SA participates in ROS-mediated SAR [10]. In the ROS/SA-mediated signal path, H_2_O_2_ signaling stimulates synthesis of SA, and SA disturbs cellular redox homeostasis by inhibiting catalase in the peroxisome [108,109]. NPR1 protein functions downstream of SA in plant immunity, sensing SA-induced cellular redox changes [110,111]. The redox-activated NPR1 translocates to the nucleus where it acts in concert with TGA transcription factors to regulate the expression of PR genes, then induces the establishment of SAR [111].

Biotic attacks also result in the rapid synthesis of JA and its receptor-active derivative, jasmonoyl-l-isoleucine (JA-Ile) [112]. JA promotes the expression of virtually all major classes of secondary metabolites and proteins that have established roles in defense, including alkaloids, terpenoids, phenylpropanoids, amino acid derivatives, anti-nutritional proteins, and some PR proteins [105]. It was found that MYC2, a basic helix-loop-helix transcription factor, has a direct role in JA-triggered immunity [113] and the two NOX genes, *AtRbohD* and *AtRbohF*, were found to be essential for the expression of the MYC2-regulated genes [114]. These results emphasize that NOXs, ROS, and associated redox processing are an integral part of hormone regulation, functioning in the control of plant immunity. Further studies on the complex network of interactions and mechanisms between the hormones and ROS will facilitate the understanding of the function of NOXs in plant immunity.

Recently, we found that the treatments of many hormones strongly influenced the expression of NOXs at the transcriptional level [22]. Many *cis*-elements, which are responsible for the treatments of different hormones such as auxin, gibberellin, ABA, ET, SA and methyl jasmonic acid (MeJA), were identified in the promoter regions of both *Arabidopsis* and rice NOX family genes. A further quantitative real-time PCR analysis showed that a very complicated expression profile exists in the NOX genes under different hormone treatments. For example, some NOX genes (*AtRbohB* and *H*) are markedly downregulated by ABA and MeJA treatments while some NOX genes (*AtRbohA, C, D, E, F* and *I*) are upregulated by these hormone treatments. In addition, the expression profiles of rice NOX genes display large tissue specificity in the shoot and root under different hormone treatments, implying the inducible complexity of the NOX genes responding to hormones.

## 5. Abiotic Stress Response May Be a Specific Plant Immunity

As discussed above, the NOX-mediated signaling strongly overlaps with the response of plants to a number of biotic and abiotic stresses. This is particularly true during stress-induced stomatal closure. Some NOXs, such as OsRbohB, AtrbohD and AtrbohF, are not only closely involved in the abiotic stress responses of plants, but also widely participate in the biotic stress responses. In addition, many studies have shown that biotic and abiotic stress factors occurring in combination may be considered interactive [115,116,117,118]. For example, an increase in wheat temperature can create a negative interactive effect by lowering resistance to bacterial, viral, fungal, and nematode pathogens [113,114,115]. In both sorghum and the common bean, drought-treated plants had a higher susceptibility to the charcoal rot fungus *Macrophomina phaseolina* [116,117]; however, drought stress enhanced the resistance of the plants to the fungus *Botrytis cinerea* in tomato [118].

Apart from the similar activation mechanism of NOXs as described above, a growing body of evidence has shown that the responses of plants during biotic and abiotic stresses have some additional similar mechanisms. A long-distance ROS-induced signaling response called systemic acquired acclimation (SAA) is considered to operate following perception of abiotic stresses, which is similar to SAR in biotic stresses. ROS is also a messenger produced in abiotic stresses activating MAPK pathways. A single MAPK cascade is involved in two or more different stress responses because of different downstream targets and thus different responses. For instance, MEKK1 is activated by ROS upon abiotic factors and biotic factors, which then activates different downstream modules, as discussed above. Despite that SA was proposed as the central component in plant immunity, it was also detected to participate widely in abiotic stress responses [109,119]. In fact, SA has been shown to improve plant tolerance to major abiotic stresses by SA-mediated control of H_2_O_2_ accumulation [119]. In addition, it was found that H_2_O_2_ can act as a downstream factor of ET signaling, conferring salinity tolerance to plants by improving Na^+^/K^+^ homeostasis in *Arabidopsis*, which is partially dependent on AtRbohF activity [120]. These findings suggest that hormones /ROS/NOXs function as an integral part involved in plant immunity, as well as in response to abiotic stresses. Hence, all these results suggest that NOX/ROS is a hub of crosstalk between different signaling pathways in resistance against both abiotic and biotic factors.

**Figure 4:**
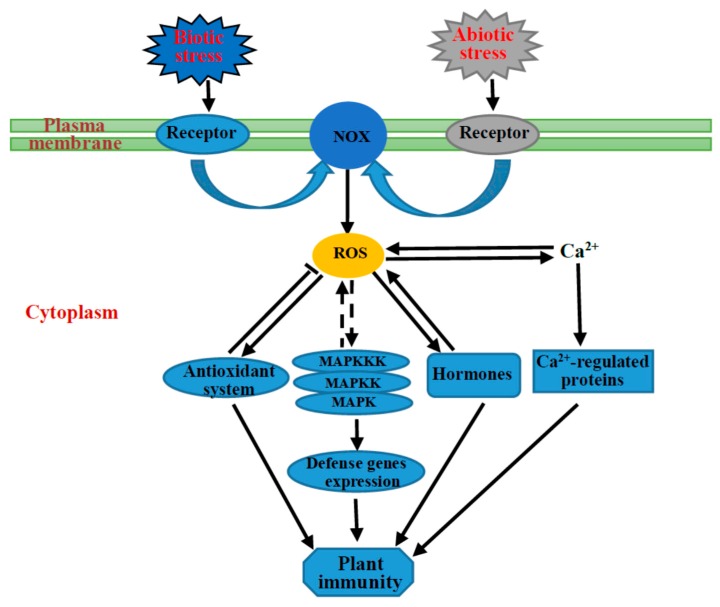
The similar regulation mechanism under biotic stress and abiotic stress responses of plants. Upon stress factor perception, the ROS produced from NOX regulate response to stress mainly through four pathways, the antioxidant system, MAPK cascades, hormones and Ca^2+^-regulated proteins. The solid lines and dashed lines represent determinate and potential interactions, respectively.

Beyond this, plants share a set of antioxidant systems when suffering from stresses whether biotic or abiotic stresses [9,121]. Any kind of biotic or abiotic stress leads to an increased level of ROS production. When the concentration of ROS is excessive, it will act as damaging [7,11,12,13]. Thus, plant stress tolerance can be improved by increasing *in vivo* levels of antioxidant systems. The plant antioxidant defense system can be divided into two parts, the non-enzymatic system and the enzymatic system. The non-enzymatic constituents include superoxide dismutase, catalase, ascorbate peroxidase, guaiacol peroxidase, glutathione reductase, glutathione peroxidase, monodehydroascorbate reductase and dehydroascorbate reductase, whereas, the pivotal non-enzymatic antioxidants include ascorbic acid, glutathione, proline, carotenoids and flavonoids [7]. It has been reported that ROS-mediated stress tolerance can be attributed to increases in the expression and activities of the antioxidant system in plants [122]. Therefore, based on the similar mechanism and common members during plant response to abiotic and biotic factors, it is reasonable to believe that the response cascade of plants to abiotic stresses is a specific plant immunity (Figure 4).

## 6. Future Perspective

In summary, NOXs function as the specific sources of apoplastic ROS, playing fundamental roles in plant immunity. The regulation of NOXs is closely associated with many signaling molecules. Although the regulation of NOXs has been described in many literature sources, the exact molecular mechanisms of regulation of NOXs and their related ROS signaling in a specific stress response are still under investigation in plants. Furthermore, the crosstalk between NOXs and protoplastic ROS production system remains to be discovered. Therefore, further work on the clarification of the aforesaid relationships is urgently required in the future.

## Abbreviations

The following abbreviations are used in this manuscript:

NOX	NADPH oxidases
RBOH	Respiratory burst oxidase homologs
ROS	Reactive oxygen species
PCD	Programmed cell death
PRR	Pattern recognition receptor
PAMP	Pathogen associated molecular pattern
BAK1	Brassinosteroid insensitive 1 associated receptor kinase 1
ETI	Effector triggered immunity
flg22	22 amino acid peptide of bacterial flagellin
GEF	Guanine nucleotide exchange factor
HR	Hypersensitive response
RACK1	Receptor for activated C-kinase 1
RLK	Receptor like kinase
RLCK	Receptor-like cytoplasmic kinase
CDPK	Calcium-dependent protein kinases
MAPK	Mitogen activated protein kinase
OST1	Open stomata 1
NADK	Nicotinamide adenine dinucleotide kinases
TFs	Transcription factors
BIK1	Botrytis-induced kinase1
FLS2	Flagellin sensing 2
EFR	Elongation factor-Tu receptor
ABA	Abscisic acid
PTI	PAMP triggered immunity
CBL	Calcineurin B-like
CIPK	CBL-interacting protein kinase
SAR	Systemic acquired resistance
ET	Ethylene
JA	Jasmonic acid
SA	Salicylic acid
PA	Phosphatidic acid
ETR1	Ethylene receptor 1
EIN2	Ethylene insensitive 2
SAA	Systemic acquired acclimation
CaM	Calmodulin
DUOX	Dual oxidases
FRO	Ferric reduction oxidase
RPS5	Resistance to pseudomonas syringae 5
CERK	Chitin-elicitor receptor kinase
CEBiP	Chitin elicitor-binding protein
